# Structural basis of transcription recognition of a hydrophobic unnatural base pair by T7 RNA polymerase

**DOI:** 10.1038/s41467-022-35755-8

**Published:** 2023-01-13

**Authors:** Juntaek Oh, Michiko Kimoto, Haoqing Xu, Jenny Chong, Ichiro Hirao, Dong Wang

**Affiliations:** 1 Division of Pharmaceutical Sciences, Skaggs School of Pharmacy & Pharmaceutical Sciences, University of California, San Diego, La Jolla, CA USA; 2 Institute of Bioengineering and Bioimaging (IBB), Agency for Science, Technology and Research (A*STAR), Singapore, Singapore; 3 Xenolis Pte. Ltd., Singapore, Singapore; 4 Department of Chemistry and Biochemistry, University of California, San Diego, La Jolla, CA USA; 5 Department of Cellular and Molecular Medicine, University of California, San Diego, La Jolla, CA USA

**Keywords:** Nucleic acids, X-ray crystallography, Structural biology, Biophysics, Transcription

## Abstract

Bacteriophage T7 RNA polymerase (T7 RNAP) is widely used for synthesizing RNA molecules with synthetic modifications and unnatural base pairs (UBPs) for a variety of biotechnical and therapeutic applications. However, the molecular basis of transcription recognition of UBPs by T7 RNAP remains poorly understood. Here we focused on a representative UBP, 7-(2-thienyl)-imidazo[4,5-b]pyridine (Ds) and pyrrole 2-carbaldehyde (Pa), and investigated how the hydrophobic Ds–Pa pair is recognized by T7 RNAP. Our kinetic assays revealed that T7 RNAP selectively recognizes the Ds or Pa base in the templates and preferentially incorporates their cognate unnatural base nucleotide substrate (PaTP or DsTP) over natural NTPs. Our structural studies reveal that T7 RNAP recognizes the unnatural substrates at the pre-insertion state in a distinct manner compared to natural substrates. These results provide mechanistic insights into transcription recognition of UBP by T7 RNAP and provide valuable information for designing the next generation of UBPs.

## Abstract (web summary)

T7 RNA polymerase (RNAP) is widely used for synthesizing RNA molecules with synthetic modifications and unnatural base pairs (UBPs). Here, authors show the structural basis of how UBPs are recognized as template and substrate, providing mechanistic insights into UBP transcription by T7 RNAP.

## Introduction

**Fig. 1:**
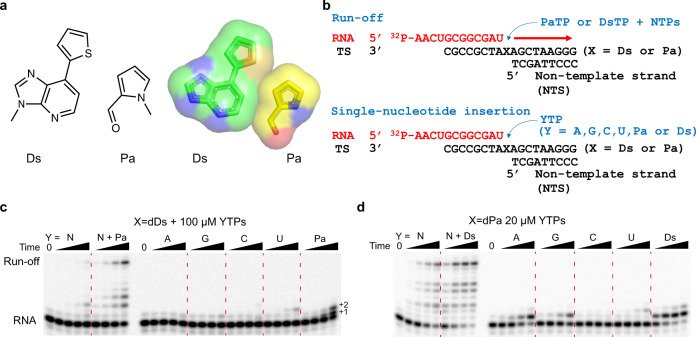
Transcription assay of Ds–Pa unnatural base pair. **a** Schematic and space filling structure of the Ds–Pa pair. **b** Biochemical assay scheme used in this study. **c**, **d** Transcription assay using unnatural bases. X indicates template-strand DNA base, while Y indicates added nucleoside triphosphate. “N” stands for all natural nucleotides, ATP, GTP, CTP and UTP (as shown in scheme). Time points were 15 sec, 1 min, 5 min and 30 min. All assays were independently repeated for three times. Source data are provided as a Source Data file.

One of the key goals in the synthetic biology field is to develop unnatural base pairs (UBPs) for expanded genetic alphabets that act orthogonally with natural base pairs in replication, transcription, and translation processes. Several groups, namely Benner, Kool, Romesberg, and Hirao groups, have designed and synthesized UBPs with distinct recognition principles from the natural Watson-Crick base pairs^1–10^. A 7-(2-thienyl)-imidazo[4,5-b]pyridine (Ds) and pyrrole 2-carbaldehyde (Pa) pair, developed by Hirao group, is a representative hydrophobic UBP that relies on shape complementarity and hydrophobic interactions, but lacks hydrogen bonding between the base pairs (Fig. 1a)^11^. The Ds–Pa pair and their derivatives can be efficiently replicated by DNA polymerases and transcribed by single-subunit bacteriophage T7 RNA polymerase (T7 RNAP) in vitro^2,12–18^.

T7 RNAP is widely used for synthesizing RNA molecules with UBP or other site-specific modifications in vitro and in vivo for a variety of biotechnical and therapeutic applications^8,9,19–25^. For example, mRNA vaccines against Covid-19 contains the modified nucleobase, N1-methylpseudouridine (m1Ψ), which is known to reduce mRNA immunogenicity and increase protein expression^26–29^. It is noteworthy that bacteriophage T7 RNAP is completely distinct from multi-subunit cellular RNA polymerases^30,31^. T7 RNAP belongs to the single-subunit right-handed polymerase superfamily, which includes almost all replicative DNA polymerases, bacteriophage single-subunit RNA polymerases (RNAPs), mitochondrial RNAPs, and reverse transcriptases^20,32^. Previous structural studies revealed the structural basis of substrate selection and nucleotide addition cycle by T7 RNAP for natural base pair system^19,21^. A striking feature is that T7 RNAP makes initial substrate selection at the pre-insertion state, where the incoming ATP substrate establishes hydrogen bonds with the template dT base before both template base and substrate are fully loaded at the catalytic center of T7 RNAP. At this stage, ATP substrate is bound along with open O-helix around 10 Å from the active center, template +1 base is sequestered in a protein pocket formed by O/O′-helices (away from the active center), and the gate residue Tyr639 is stacked with upstream template −1 base occluding the template +1 base loading. In addition to nucleobase selection via hydrogen-bonding base-pairing, T7 RNAP selects rNTP over dNTP through Mg^2+^-mediated interaction of Tyr639 (Y639) residue with 2′-OH of substrate ribose at the pre-insertion state. Therefore, the pre-insertion state represents a critical transcription fidelity checkpoint for T7 RNAP transcription: the mismatched substrate can be effectively rejected at the pre-insertion state and cannot go to the closed state for insertion. Selection of correct substrate over mismatched substrate at the pre-insertion state (before O-helix conformational change) is also suggested to be energetically more efficient than the selection at the insertion state (after O-helix conformational change)^33^. Upon the correct substrate binding at the pre-insertion state, T7 RNAP undergoes coordinated conformational changes in order to proceed to the insertion state for chemical reaction. These coordinated movements include the loading of template +1 base and incoming NTP to the active center, the displacement of blocking Y639, and the rotation of the O-helix subdomain to seal off the active site. The substrate selection specificity by T7 RNAP at pre-insertion state requires a complementary hydrogen-bonding pattern among the Watson-Crick base pairs. Given that hydrophobic Ds–Pa pair lacks hydrogen bonding, it raises an intriguing unanswered question: what is the molecular mechanism of Ds–Pa pair recognition by T7 RNAP? This represents a critical knowledge gap in the field.

In this work, we investigate the molecular mechanism of Ds–Pa pair transcription recognition by T7 RNAP using a combined approach including enzyme kinetics, structural biology, modeling, and mutagenesis. We performed kinetic analysis to investigate how T7 RNAP elongation complex selectively recognizes dDs or dPa template and incorporates corresponding unnatural nucleotide substrates (PaTP or DsTP) over natural nucleotides during transcription. To understand the structural basis of UBP transcription, we solved six T7 RNAP–UBP complex structures with or without its cognate substrates (dDs, dPa apo structures, dDs–PaTP, dPa–DsTP, dDs–ATP, dPa–ATP complex structures). Our results showed that both dDs and dPa prefers its unnatural pairing partner (with variations in incorporation efficiency and fidelity). We observed a unique unnatural nucleoside triphosphate binding mode for PaTP and DsTP, suggesting distinct substrate recognition mechanism of UBP during T7 RNAP elongation. Guided by structures, we further identified a separation-of-function mutant of T7 RNAP that does not affect natural nucleic acids transcription, but selectively modulates UBP transcription. Taken together, these results provide mechanistic insights into UBP transcription and potential design strategy for the next generation of UBPs.

## Results and discussion

### Selective transcription recognition of Ds–Pa by T7 RNAP

Previous studies suggested that Ds–Pa pair can be transcribed by T7 RNAP^11,13–16^. However, the previous promoter dependent transcription system is not suitable for detailed kinetic studies, and it is also difficult to dissect the transcription initiation and elongation phases. To understand the recognition of Ds–Pa pair and substrate selectivity by T7 RNAP at elongation phase, we assembled T7 RNAP elongation complex with synthetic scaffolds^34^. This system allowed us to perform single-nucleotide incorporation assay for T7 RNAP elongation complex on UBP-containing scaffolds (Fig. 1b–d). We found that T7 RNAP can recognize both dDs and dPa templates and preferentially insert their designed substrate, PaTP and DsTP, respectively. Specifically, PaTP is the only substrate to be incorporated opposite to the dDs template (Fig. 1c). Consistently, PaTP is required for T7 RNAP to bypass the Ds site and produce the run-off transcript during transcription elongation. In contrast, for the dPa template, we observed rapid DsTP addition and slow addition for ATP and GTP (with longer incubation time) (Fig. 1d).

To provide a quantitative measurement of substrate selectivity, we then performed pre-steady-state single-turnover transcription assays. The kinetic parameters, *k*_pol_ (catalytic rate constant) and *K*_d_app_ (apparent dissociation constant), for PaTP, DsTP, and ATP incorporation were determined using the dDs, dPa, and dT templates, respectively (Supplementary Fig. 1 and Table 1). It is noteworthy that the *k*_pol_ of DsTP incorporation opposite dPa template is comparable to that of ATP incorporation opposite dT template, whereas the overall *K*_d_app_ value of DsTP for dPa template (40 μM) is around 500-fold higher than that of ATP for dT template (0.08 μM). As a result, the incorporation efficiency of DsTP for Pa template (determined by specificity constant *k*_pol_/*K*_d_app_) is about 540-fold less than that of ATP incorporation for dT template, where the difference is mainly caused by *K*_d_app_ value instead of *k*_pol_ value. Interestingly, transcription recognition efficiency of the Ds–Pa pair by T7 RNAP is asymmetric. The incorporation efficiency of DsTP for dPa template is about 150-fold higher than the PaTP incorporation for dDs template. This is due to a combination of ~8-fold tighter binding of DsTP over PaTP (measured by *K*_d_app_) and ~18-fold faster for DsTP incorporation over PaTP incorporation (measured by *k*_pol_).

**Table 1:** Kinetic parameters of dT–ATP, dDs–PaTP, dPa–DsTP and dPa–ATP incorporation

	*k*_*pol*_ (min^−1^)	*K*_*d_app*_ (μM)	*k*_*pol*_ /*K*_*d_app*_ (min^−1^ μM^−1^)	Relative efficiency
dPa–DsTP	460 ± 10	40 ± 4	12 ± 1	1
dT–ATP	500 ± 20	0.08 ± 0.01	(6.2 ± 0.9) * 10^3^	540 ± 80
dDs–PaTP	25 ± 2	330 ± 70	(7.6 ± 1.7) * 10^−2^	(6.5 ± 1.5) * 10^−3^
dPa–ATP	130 ± 10	420 ± 50	0.31 ± 0.04	(2.7 ± 0.4) * 10^−2^

*K*_d_app_, *k*_pol_, incorporation efficiency as *k*_pol_/*K*_d_app_, and relative efficiency are shown.

Comparison of kinetic parameters of DsTP and the mismatched natural nucleotide (ATP) incorporation provides a quantitative measurement of nucleotide selectivity, or discrimination. For the dPa template, we found ~10.5-fold tighter binding of DsTP over ATP (measured by *K*_d_app_) and ~3.5-fold faster for DsTP incorporation over ATP incorporation (measured by *k*_pol_). Taken together, the overall discrimination of DsTP over ATP is about 37-fold, suggesting T7 RNAP preferentially binds and incorporates DsTP over ATP.

### UBP template recognition in T7 RNAP elongation complex

To understand how Ds–Pa UBPs are recognized and processed by T7 RNAP, we solved six different structures of T7 RNAP elongation complexes containing a site-specific dDs or dPa at the +1 position of the template strand (Supplementary Table 1).

**Fig. 2:**
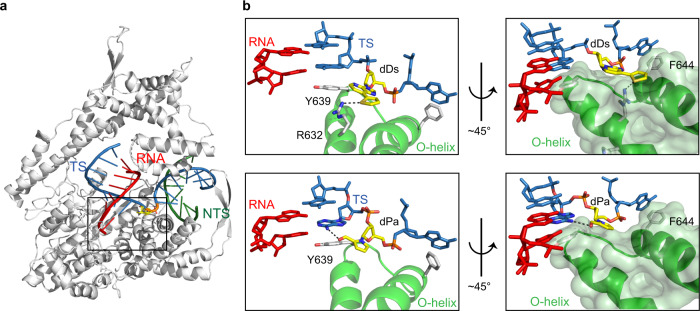
Unnatural template loading by T7 RNA polymerase. **a** Overall structure of T7 RNA polymerase. Template-strand DNA, RNA and non-template-strand DNA are colored blue, red, and green, respectively. **b** Active site of apo dDs or dPa harboring T7 RNAP (left panel). Right panel shows surface of O-helix and UBP together to indicate fitting of UBP above the O-helix. O-helix and its surface is colored in green. +1 template base is colored in yellow. Other interacting residues are colored in white. Potential hydrogen bonding distance between UBP and T7 RNAP residues are labeled with black dash.

We first focused on the recognition of dDs or dPa template base by T7 RNA polymerase. To make a direct comparison with natural T7 RNAP elongation complex^35,36^, we adopted the experimental method and the same DNA/RNA hybrid scaffold (except the +1 position at the template strand) from previously reported natural T7 RNAP elongation complex. We crystallized T7 RNAP containing a site-specific dDs or dPa at the +1 position of the template strand DNA. The overall structures of UB-containing T7 RNAP elongation complexes are essentially identical to that of natural T7 RNAP (Fig. 2a)^35^. T7 RNAP is captured at the post-translocation state, where the elongation complex is waiting for incoming substrate and gating Y639 is stacking with the −1 template-strand base. Both dDs and dPa are accommodated at a binding pocket over the O-helix, essentially the same as the natural bases (Fig. 2b and Supplementary Fig. 2)^35^. Intriguingly, we observed UB-specific interactions between the unnatural base and T7 RNAP. The dDs base fits perfectly to the surface of the O-helix and appears to interact with R632 via hydrogen bonding between the sulfur atom in Ds and the carboxyl group in R632. We also observed the aldehyde group of dPa base at +1 position forms hydrogen bonding with 6-amine group of adenine base at the −1 position, suggesting certain nucleobase of flanking sequence may contribute to stabilize Pa conformation during the template loading.

### UBP substrate recognition by T7 RNAP at pre-insertion state

Previous studies revealed that the cognate natural nucleotide forms hydrogen bonds with its corresponding template base at the pre-insertion state, a critical fidelity checkpoint state before T7 RNAP proceeds into the insertion state and commits to nucleotide incorporation^19,21^. At the pre-insertion state, the incoming nucleotide substrate establishes initial hydrogen bonding and base pairing with the +1 template base, and the gate residue Y639 remains stacking with −1 base. A key feature of pre-insertion state is that the +1 base pair (+1 template base and incoming substrate) is not yet fully loaded to establish base stacking interactions with upstream RNA:DNA hybrid, and the O-helix remains in an open conformation.

**Fig. 3:**
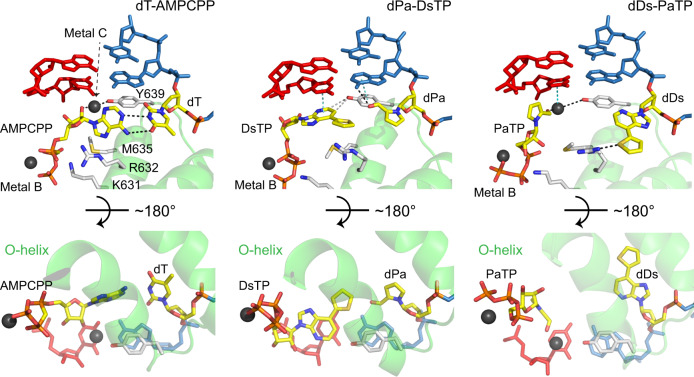
Comparison of pre-insertion state between natural and unnatural base pairs (UBPs). Pre-insertion state with dT–AMPCPP (PDB code: 1S76), dPa–DsTP and dDs–PaTP pair. Key residues for recognizing incoming nucleoside triphosphate as well as template strand are shown in white. +1 template base and substrate are shown in yellow. Magnesium ions (Metal B: interacts with triphosphate moiety. Metal C: interacts with Y639) are colored in dark gray. Hydrophilic and stacking interactions are shown in black and cyan dash.

Because the Ds–Pa pair lacks hydrogen bonding but still supports faithful transcription in vitro, we are interested in understanding how an incoming unnatural nucleoside triphosphate is recognized by T7 RNAP. By soaking with their cognate or non-cognate substrate, PaTP/ DsTP or ATP, we got four T7 RNAP substrate-bound structures; dDs–PaTP, dDs–ATP, dPa–DsTP and dPa–ATP structures (Fig. 3 and Supplementary Figs. 3–5). Intriguingly, we observed that both DsTP and PaTP substrates adopt unique conformations that are not observed in previous T7 RNAP structures with natural substrate^21^. The substrate recognition patterns of DsTP and PaTP are therefore very distinct from their natural nucleotide counterpart.

Previous studies revealed that the recognition of natural ATP at the pre-insertion state is achieved by three layers of key interactions^21^. First, the base moiety of incoming NTP forms hydrogen bonds with +1 template base. Second, the ribose moiety of substrate is recognized by R632 and magnesium ion (metal C)—Y639 bridging. Finally, the triphosphate moiety together with the magnesium ion (metal B) are recognized by D471, K472, R627, and K631. In sharp contrast, both DsTP and PaTP only maintain key interactions to hold magnesium ion (metal B) and its triphosphate moiety (Fig. 3 and Supplementary Figs. 3, 4).

For the T7 RNAP containing dPa–DsTP, we captured a distinct intermediate state between the pre-insertion state and insertion state, termed “primed” state. In this state, DsTP is shifted more toward the binding site at the insertion state. The expanded nucleobase of DsTP is able to stack with the nucleobase of 3’-end of the RNA. In the primed state, Y639 still maintains stacking with −1 template base. The shape of Ds aromatic ring is complementary to the benzene ring of Y639 and makes perfect edge-to-edge base pair interaction with Y639 and stacking with −1 RNA:DNA base pair (Fig. 3).

For the T7 RNAP containing dDs–PaTP structure, we observed that ribose and base moieties of PaTP rotated ~180 degree while maintaining magnesium ion (metal B) and triphosphate moiety interactions. As a result of ribose moiety rotation, we found that R632 interacts with the sulfur atom of template dDs instead of the O4 atom of the ribose ring of nucleotide substrate. K631, instead of R632, interacts with both 3′ hydroxyl group and phosphate group of PaTP (Fig. 3). Intriguingly, we also observed additional density near aldehyde group of Pa nucleobase. Given the published pre-insertion state structure on the natural scaffold had a Mg^2+^ ion (metal C) at the similar position (Fig. 3a) and short distance between the peak of density and aldehyde group of Pa (around 2.1 Å), we modeled Mg^2+^ ion as putative metal C^21^. Due to moderate resolution of structure, we did not rule out an alternative possibility that this density can also be Na^+^ ion or a water molecule. Nevertheless, this putative metal C could serve as a hub for a potential interaction network bridging together the incoming PaTP, gate Y639 residue, and 3′-RNA primer (Fig. 3 and Supplementary Fig. 3).

Our structures of pre-insertion state complexes reveal unique pre-insertion binding sites for DsTP and PaTP, shedding light on the molecular mechanism of nucleotide recognition of hydrophobic UBP without hydrogen-bonding interactions between the pairing bases. We found that DsTP is stabilized by its strong stacking interaction with 3′ RNA primer, highlighting the functional importance of expanded planar thienyl group (in addition to its steric hindrance to prevent mispairing with other natural nucleotides). DsTP is much closer to the active center than canonical pre-insertion binding site. This is consistent with the kinetic study, which shows high *k*_pol_ value of dPa–DsTP incorporation, indicating that once the substrate is recognized, the nucleotide addition will occur efficiently. We also found the PaTP is likely restrained by the specific interactions among its aldehyde group, metal ion, and RNAP residues. The pre-insertion binding site of PaTP requires larger conformational movement to the active center, which is consistent with slow kinetics and low *k*_pol_ value of PaTP incorporation.

To understand mismatched incorporation, we also solved the structures of T7 RNAP complexes containing dPa–ATP and dDs–ATP (Supplementary Figs. 4, 5). In the case of dPa–ATP structure, we observed a defined density that allows us to model ATP at the active site. The base moiety of ATP was stacking with 3’ RNA, but it was flipped ~180 degrees to point its amine group toward the minor groove of DNA/RNA hybrid. Therefore, ATP needs to be flipped 180 degrees to allow nucleotide addition, which may explain why incorporation efficiency of ATP toward dPa is much lower than that of DsTP (Supplementary Fig. 5 and Table 1). Intriguingly, in the dDs–ATP complex structure, only triphosphate moiety of ATP is visible, where the density map for ribose and base moieties is weak and discontinuous (Supplementary Fig. 4). This is consistent with our biochemistry results that almost no ATP addition was observed against dDs template.

### M635 as a key residue for UBP transcription

**Fig. 4:**
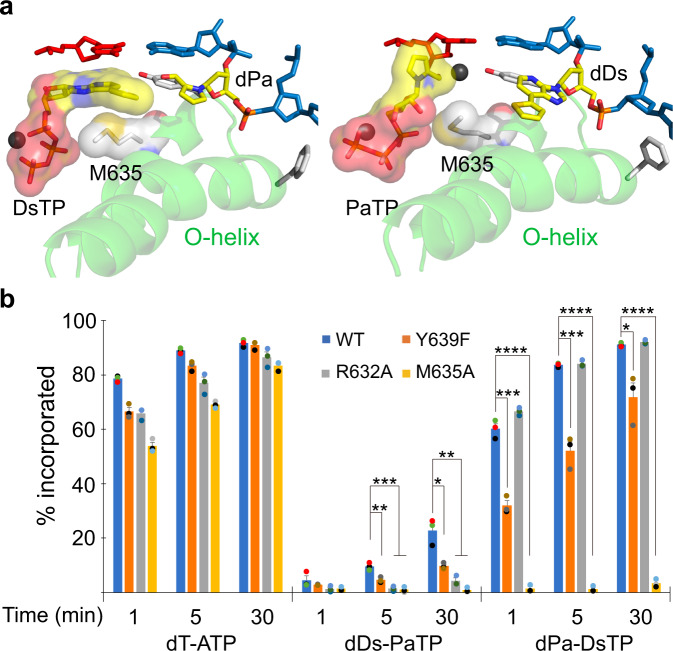
Single-nucleotide incorporation analysis of T7 RNAP mutants. **a** Surface representation of incoming DsTP or PaTP and M635. Surface is colored by atom type. Carbon of M635 and DsTP are colored as white and yellow, respectively. **b** Relative single-nucleotide incorporation efficiency of T7 RNAP WT and mutants. Substrate concentration was 100 μM. Data are presented as mean values ± SEM. All data in this figure were obtained and quantified from three independent experiments (*P < 0.05; **P < 0.01; ***P < 0.001; ****P < 0.0001, two-tailed Student’s t-test). Each data point is represented by a dot. *P* values of each comparison are: 0.0066 and 0.0102 for dDs–PaTP, WT vs. Y639F, 5 and 30 min. 0.00094 and 0.0034 for dDs–PaTP, WT vs. R632A, 5 and 30 min. 0.00097 and 0.00155 for dDs–PaTP, WT vs. M635A, 5 and 30 min. 0.00045 and 0.00075 and 0.022 for dPa–DsTP, WT vs. Y639F, 1 and 5 and 30 min. 0.000009 and 0.00000004 and 0.00000007 for dPa–DsTP, WT vs. M635A, 1 and 5 and 30 min. Source data are provided as a Source Data file.

As we examined our UBP-containing T7 RNAP complex structures, we noticed several key residues that behave differently compared to natural pre-insertion state. Y639, which was interacting with 2’ hydroxyl group in natural base pair transcription, now interacts with the aldehyde group of PaTP via magnesium ion (and close distance to DsTP). Previous studies reported that, for the Y639F mutant, discrimination power of rATP over dATP selection decreases over 20-fold (from ~120–140 fold for WT to ~5–7 fold for Y639F)^37,38^. R632, which was interacting with oxygen of ribose, now interacts with the sulfide group of dDs. M635 seems to provide a hydrophobic effect to stabilize incoming substrate. To dissect their roles in UBP transcription, we generated T7 RNAP mutants with a single-residue substitution, Y639F, R632A or M635A, as well as M635K and tested their single-nucleotide incorporation efficiency (Fig. 4 and Supplementary Fig. 6).

Intriguingly, we observed very distinct patterns of these substitutions. As shown in Fig. 4, Y639F substitution leads to similar modest decreased activities in all scaffolds we tested. Interestingly, R632A substitution results in a stronger inhibitory effect on PaTP incorporation than that for DsTP or natural ATP incorporation. R632A mutant leads to more than 5-fold inhibition for dDs–PaTP incorporation. Most strikingly, M635A substitution completely abolishes UBP incorporation (dDs–PaTP and dPa–DsTP), whereas it has no effect on natural ATP incorporation against dT template. Interestingly, we also found that M635K substitution also completely abolishes UBP incorporation (Supplementary Fig. 6). We reasoned that while the shorter side chain (A vs M) of M635A compromises its direct contact with UBP substrate, the longer side chain (K vs M) of M635K mutant may cause steric clash with UBP during incorporation. This result highlights that T7 RNAP residues may have a distinct role in mediating transcription from UBP scaffolds versus transcription from natural scaffolds. In particular, we found that M635 becomes dominantly important for Ds–Pa pair transcription (Fig. 4a).

### Modeling the UBP insertion state

Upon initial substrate recognition at the pre-insertion state, Y639 needs to be rotated out to allow the full-loading of substrate and +1 template base to establish stacking with the upstream base pair of RNA:DNA hybrid (−1 base pair). The O-helix is rotated about 22 degrees to the active site to form a closed complex for nucleotide addition (insertion state). We do not have a crystal structure of this closed complex (insertion state) currently. To gain insights into how dDs–PaTP and dPa–DsTP pairs are accommodated at the insertion site, we generated insertion-state models for UBP-containing T7 RNAP complex by superimposing a planar Ds–Pa pair. This model was generated by combining previous structural studies on KlenTaq DNA polymerase harboring dDs in template strand and incoming dPxTP (PDB code: 5NKL) and T7 RNAP natural elongation complex with natural NTP at insertion state (PDB code: 1S76) (Supplementary Fig. 7 and Supplementary Movie 1, see method for model building)^19,39^. The Ds–Px pair was developed for high fidelity UBP replication than that of Ds–Pa. The oxygen in the nitro group of Px repels the 1-nitrogen of A, and thus Px prevents pairing with A^12,17,18^. In these models, the Ds–Pa base pair fit nicely within T7 RNAP active site without obvious steric clash. This suggests after being recognized by unique pre-insertion state, these UBPs may adopt a similar insertion state as a natural base pair does. The Ds–Pa planar conformation can be well accommodated by T7 RNAP active site. We also noticed that T7 RNAP dDs–PaTP complex undergoes more conformational change than dPa–DsTP to form the closed state. This is consistent with our kinetic studies, showing that the incorporation of DsTP opposite dPa is much faster than PaTP opposite dDs.

### Mechanistic insight of UBP transcription by T7 RNA polymerase

**Fig. 5:**
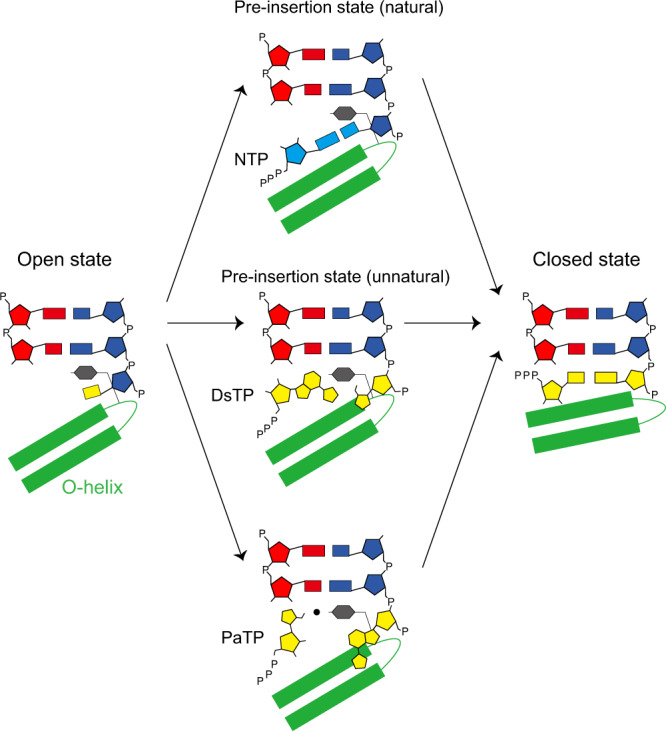
Proposed mechanism of UBP transcription by T7 RNA polymerase. dDs and dPa can be loaded above the O helix, with essentially identical conformation to that of natural template base. However, due to a lack of hydrogen bonding, incoming substrate cannot adopt pre-insertion state as natural base pairs do, where hydrogen bonding-based recognition is critical. Instead, each DsTP and PaTP adopts a unique substrate binding configuration, which utilizes base stacking and magnesium-pi stacking to be stabilized in the active site, while maintaining interaction to hold triphosphate, magnesium ion, and ribose moiety. Our structural model and kinetics assay suggest once substrate is bound, elongation complex will commence conformational change to closed state and allow addition of substrate. Yellow boxes in open state or closed state indicates universal base which can be natural or unnatural bases.

Here we provide the structural basis of transcription recognition and nucleotide selection of UBP (Ds–Pa) by T7 RNA polymerase. We solved the six T7 RNAP–UBP elongation complex structures at post-translocation state and substrate-bound pre-insertion state. Based on these structural studies, together with biochemical and modeling results, we propose a mechanism of Ds–Pa transcription by T7 RNA polymerase (Fig. 5). First, in the post-translocation state (apo), the dDs or dPa template base can be easily loaded at the pocket above O-helix with similar conformation to that of natural bases. We observed additional specific interactions to recognize these unnatural bases. Second, we observed DsTP or PaTP adopts a unique pre-insertion state, which was not observed in any other natural substrate structures. Although the pre-insertion states of UBP are different from those of natural pairs, our biochemical assays suggest that these pre-insertion states can readily allow specific nucleotide addition reaction. Based on molecular modeling, we predict that Ds–Pa pair can adopt similar and stable planar structure at insertion site as observed in KlenTaq DNA polymerase. Future studies will focus on understanding the structural details of transition from the pre-insertion state to other states (insertion state, product state, etc) to fully understand the nucleotide addition cycle of the Ds–Pa pair.

## Methods

### Preparation of unnatural oligonucleotides and nucleoside triphosphates

18-mer DNA templates (5′-GGGAATCGAXATCGCCGC, X = Ds or Pa) containing unnatural bases were chemically synthesized with an H8 DNA/RNA Synthesizer (K&A Laborgerate), using phosphoramidites reagents for the natural and Ds or Pa bases, followed by purification with denaturing gel electrophoresis. The unnatural nucleotides, Ds and Pa phosphoramidites, and Ds and Pa triphosphates (DsTP and PaTP), were prepared as described previously^11^.

### Purification and crystallization of T7 RNA polymerase and mutants

Wild-type T7 RNAP was cloned into modified pET28 vector harboring N-terminal 6-histidine tag, followed by TEV protease recognition site. The plasmid was transformed into Rosetta 2 (DE3) and the T7 RNAP expression was induced by IPTG at OD_600_ of 0.6, followed by overnight incubation at 20 °C. First purification was performed by using Ni-NTA agarose (Qiagen), following manufacturer’s instruction. Briefly, cells were collected, resuspended, and disrupted by microfluidizer in buffer A: 20 mM HEPES pH 8.0, 300 mM NaCl, 5% glycerol and 2 mM β-mercaptoethanol (BME). After lysis, centrifugation followed to remove pellets. Supernatant was loaded onto the Ni-NTA column and washed with buffer A supplemented with 20 mM imidazole (A + 20 mM imidazole buffer) and buffer A supplemented with 30 mM imidazole (buffer A + 30 mM imidazole buffer). On-column TEV protease digestion was carried out at 4 °C overnight to remove the 6-His tag. After cleavage, tag-free T7 RNAP was eluted with A + 30 mM imidazole buffer. Eluted sample was further purified by using Heparin HP column (Cytiva). Purified T7 RNAP was concentrated to 401 μM in buffer B: 20 mM HEPES pH 8.0, 200 mM NaCl, 5 mM BME and 20 mM MgCl_2_. WT pQE-T7 was then mutated to Y639F, R632A, or M635A, respectively. Each single-mutant T7 plasmid was transformed into BL21 (non-DE3) to ensure WT-T7 RNAP free expression and purification. His-tagged proteins were purified by Ni-NTA and Heparin column without removing tag.

Crystallization of T7 RNAP was performed as previously described^36^. Briefly, mini-scaffold was prepared by annealing 18-mer template-strand DNA (5′-GGGAATCGAXATCGCCGC, X = Ds or Pa), non-template DNA (5′-TCGATTCCC) and RNA (5′-AACUGCGGCGAU) at 1:1:1.2 molar ratio in a buffer containing 10 mM Tris pH 8.1, 200 mM NaCl, 20 mM MgCl_2_ and 5 mM BME. For dDs apo, dPa apo and dDs–PaTP structures, natural RNA with 3′ hydroxyl group was used. For dDs–ATP, dPa–DsTP and dPa–ATP structures, 3′ deoxy RNA (to avoid nucleotide reaction) was used to improve diffraction quality. T7 RNAP elongation complex was formed by mixing protein and scaffold at 1:1.2 molar ratio. Subsequently, 10 mg/ml of T7 RNAP elongation complex was crystallized by mixing same volume of crystallization buffer (100 mM Tris pH 8.1, 10–14% PEG 8000, 8% glycerol and 5 mM BME) in hanging drop vapor diffusion plate at 22 °C.

### Structure determination of T7 RNA polymerase

X-ray datasets were collected at BL12-2, Stanford Synchrotron Radiation Lightsource, SLAC National Accelerator Laboratory (for dDs–PaTP and dDs apo datasets) and BL 5.0.1 (for dPa apo, dPa–ATP and dDs–ATP dataset) and 8.2.1 (for dPa–DsTP dataset), Advanced Light Source, Lawrence Berkeley National Laboratory, respectively. Collected images were processed by XDS (built = 20190417) using CC1/2 higher than 0.3 to determine high resolution cutoff^40^. One exception was dDs apo structure, which shows low completeness when single crystal is processed by XDS. To overcome this problem, we processed and merged two datasets from two independent crystals, using xia2/dials (version 3.8.0) in ccp4i program suite^41^. This improved overall completeness from 88% to 97% (Supplementary Table 1, dDs_apo section). Space group of T7 RNAP–UBP crystals were P1, with four elongation complexes in the asymmetric unit. Molecular replacement was done for phasing, using chain B in the PDB 1H38 as search model^35^. Several rounds of manual building and refinements were performed by using Phenix (version 1.19) and COOT (version 0.9.8.2) to get the final refined structure^42,43^. Data collection and refinement statistics are summarized in Supplementary Table 1. Figures containing structures are prepared by Pymol (version 2.5).

### In vitro transcription assay

Transcription assay was performed with the same scaffold used for crystallization. Mini-scaffold was prepared by annealing template-strand DNA, non-template-strand DNA, and P^32^-labeled RNA at 2:3:1 molar ratio in elongation buffer (20 mM Tris pH 7.5, 40 mM KCl, 5 mM MgCl_2_, and 5 mM DTT). Annealing was performed by heating the scaffold mixture at 80 °C for 5 min and cooling down to room temperature. Transcription assay method was essentially identical as described before^44^. Briefly, the final reaction mixture contains 20 nM mini-scaffold, 120 nM T7 RNA polymerase, and varying concentration of NTP, PaTP or DsTP in elongation buffer. At each time point, 1.5 μl of the reaction mixture was added to 6 μl of stop buffer (90% formamide, 10% 0.5 M EDTA pH 8.0 with bromophenol blue and xylene cyanol dyes). After the reaction, all samples were denatured at 95 °C for 10 min and analyzed by Urea/TBE PAGE. For kinetic analysis, initial velocity and *K*_d_app_ (apparent dissociation constant) was calculated by fitting to Michaelis-Menten model, using Prism regression software (version 8). All images were quantitated by using Image Lab software (version 6.0.1).

### Reporting summary

Further information on research design is available in the Nature Portfolio Reporting Summary linked to this article.

## Supplementary information

Supplementary Information

Description of Additional Supplementary Files

Supplementary Movie 1

Reporting Summary

## Data Availability

The data that support this study are available from the corresponding authors upon reasonable request. Coordinates have been deposited in the Protein Data Bank (PDB) under accession codes 8DH0 (dDs apo), 8DH1 (dDs–PaTP complex), 8DH2 (dDs–ATP complex), 8DH3 (dPa apo), 8DH4 (dPa–DsTP complex), and 8DH5 (dPa–ATP complex). Other PDB structures referred in this study are 5NKL, 1S76 and 1H38. Source data are provided with this paper.

## Source data

Source Data
