# Supplementary material for: Structural basis of transcription recognition of a hydrophobic unnatural base pair by T7 RNA polymerase
Source: Nat Commun. 2023 Jan 13;14:195. doi: 10.1038/s41467-022-35755-8 (PMC9836923; doi:10.1038/s41467-022-35755-8)
Supplement: Supplementary file 1 — Supplementary Information [file 41467_2022_35755_MOESM1_ESM.pdf]

Supplementary information for

Structural Basis of Transcription Recognition of a Hydrophobic Unnatural Base Pair by T7 RNA Polymerase

Juntaek Oh[1], Michiko Kimoto[2,3], Haoqing Xu[4], Jenny Chong[1], Ichiro Hirao[2,3,*], Dong Wang[1,4,5,*]

[1]Division of Pharmaceutical Sciences, Skaggs School of Pharmacy & Pharmaceutical Sciences; University of California, San Diego, La Jolla, California 92093, United States

[2]Institute of Bioengineering and Bioimaging (IBB), Agency for Science, Technology and Research (A*STAR), 31 Biopolis Way, The Nanos, #07-01, Singapore 138669, Singapore

[3]Xenolis Pte. Ltd., 79 Science Park Drive, #06-01/08, Cintech IV, Singapore 118264, Singapore

[4] Department of Chemistry and Biochemistry, University of California, San Diego, La Jolla, California 92093, United States

[5] Department of Cellular and Molecular Medicine, University of California, San Diego, La Jolla, California 92093, United States

[*] To whom correspondence should be addressed. Tel: +1 858 822 5561; Fax: +1 858 822 1953; Email: DW: dongwang@ucsd.edu; IH: ichiro.hirao@xenolis.com

DW: https://orcid.org/0000-0002-2829-1546, IH: https://orcid.org/0000-0002-1115-8079, MK: https://orcid.ogr/0000-0002-6261-7562

Supplementary Table 1. Structural data collection and refinement statistics.

	dDs apo	dDs PaTP	dDs ATP	dPa apo	dPa DsTP	dPa ATP
PDB ID	8DH0	8DH1	8DH2	8DH3	8DH4	8DH5
**Data collection**						
Space group	P 1					
a, *b*, *c* (Å)	78.7 86.2 201.2	78.9 86.2 201.7	78.7 85.0 200.5	79.1 86.3 202.0	78.8 86.2 201.5	78.5 86.3 201.2
α, β, γ (º)	89.9 85.2 69.5	89.8 85.1 69.6	89.9 85.4 69.5	89.7 86.0 69.5	89.9 85.4 69.6	89.8 85.4 69.5
Resolution (Å)*	42.0 - 2.9 (3.0 - 2.9)*	39.34 - 2.65 (2.74 - 2.65)	47.4 - 2.9 (3.0 - 2.9)	46.9 - 3.0 (3.1 - 3.0)	43.2 - 2.8 (2.9 - 2.8)	46.7 – 2.85 (2.95-2.85)
Unique reflections	107551 (10843)	130163 (12290)	106218 (10524)	98592 (9804)	119215 (11901)	113728 (11343)
Multiplicity	7.0 (6.9)	3.9 (3.9)	3.6 (3.7)	3.6 (3.7)	3.8 (3.9)	3.6 (3.5)
Completeness (%)	96.9 (87.9)	89.6 (85.5)	97.9 (96.5)	98.3 (97.6)	97.7 (97.6)	98.4 (97.1)
Mean I/sigma(I)	6.9 (0.5)	9.3 (0.4)	9.1 (0.9)	10.1 (0.9)	8.2 (0.6)	9.8 (0.9)
R-merge	0.13 (1.03)	0.17 (1.72)	0.15 (1.76)	0.11 (1.60)	0.08 (1.63)	0.12 (1.62)
CC1/2	1.0 (0.83)	0.98 (0.35)	0.99 (0.34)	1.0 (0.39)	1.0 (0.29)	1.0 (0.34)
**Refinement**						
No. reflections	107551 (9662)	128973 (12284)	105747 (10356)	98245 (9751)	119215 (11872)	113507 (11233)
R_{work} / R_{free}	0.221 / 0.261	0.230 / 0.272	0.260 / 0.308	0.247 / 0.278	0.233 / 0.275	0.234 / 0.267
No. atoms						
Macromolecules	26077	27087	27806	28298	28446	27994
Ligands	124	120	174	72	144	198
RMS (bonds, Å)	0.004	0.004	0.004	0.003	0.004	0.005
RMS (angles, °)	0.79	0.70	0.85	0.67	0.78	0.79
Clashscore	9,87	5.98	8.15	7.70	6.77	7.04
Average B-factor	101.14	100.54	123.91	136.66	116.82	108.69
Macromolecules	99.46	98.01	121.49	131.53	113.15	102.74
Ligands	96.44	89.25	121.86	127.19	108.56	104.51

*Values in parentheses are for the highest-resolution shell.

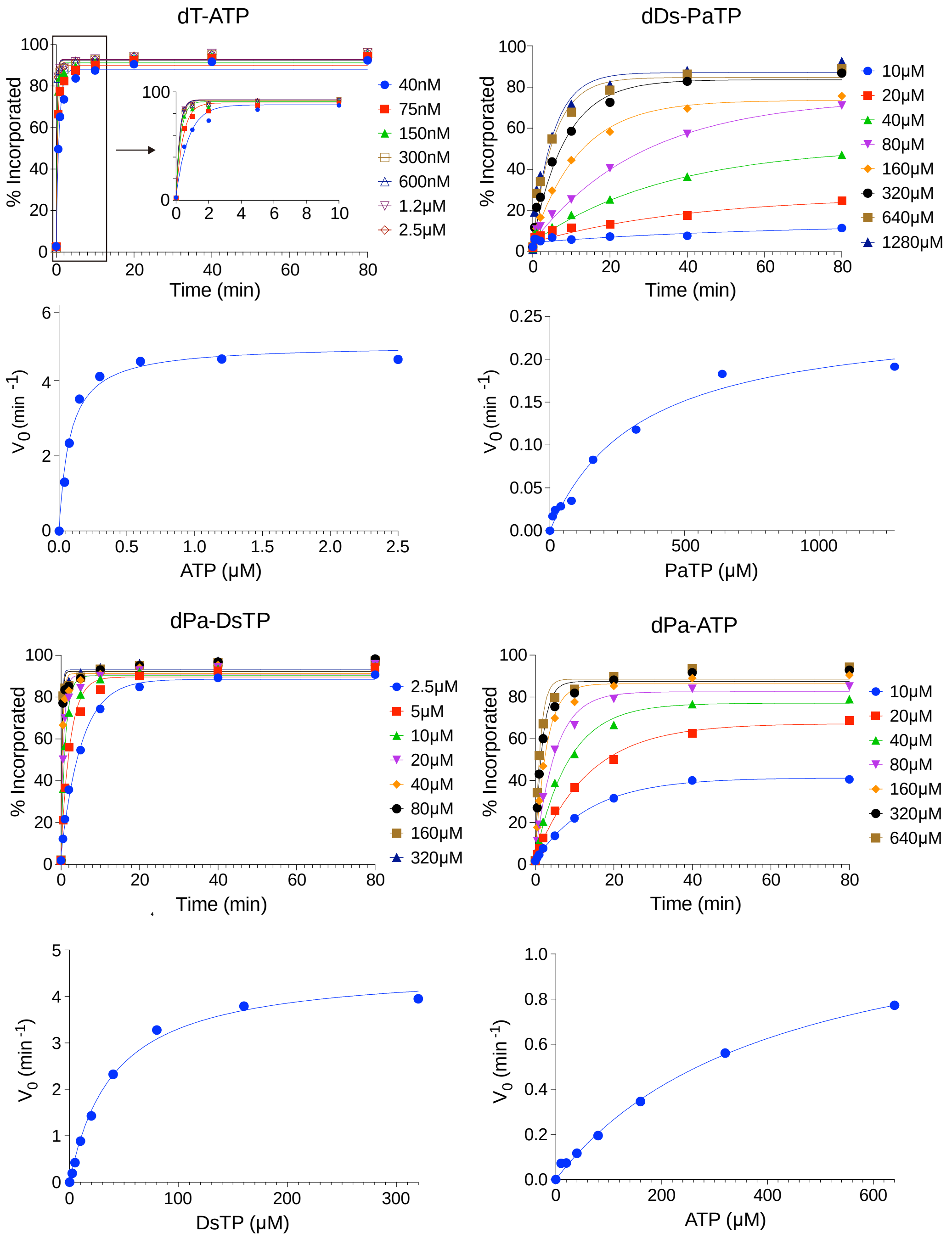

Supplementary Figure 1. Kinetic analysis of natural and unnatural base pair incorporation. Kinetic parameters were determined by single-nucleotide incorporation assays, with varying concentration of substrate and incubation time from 30 sec to 80 min. Regression curve was generated by using Prism. Source data are provided as a Source Data file, n=1.

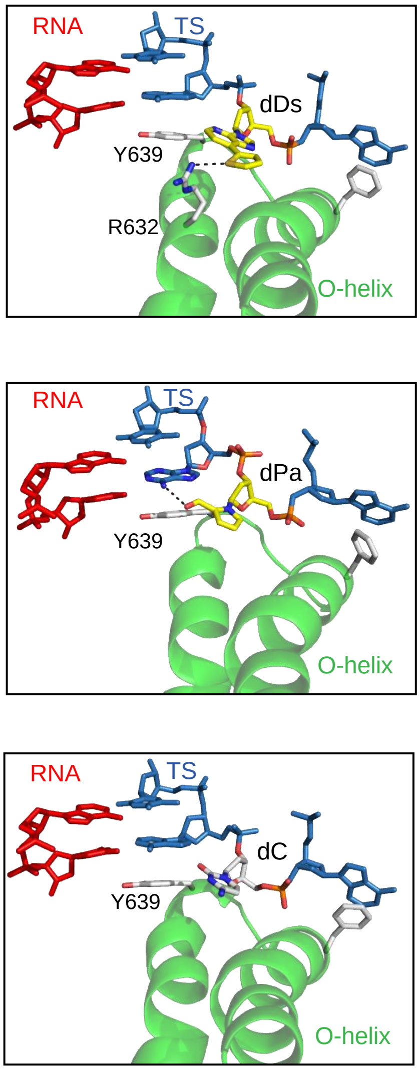

Supplementary Figure 2. Comparison of dPa and dC template loading by T7 RNA polymerase. Apo T7 RNAP elongation complex structure harboring dC template (PDB code: 1H38).

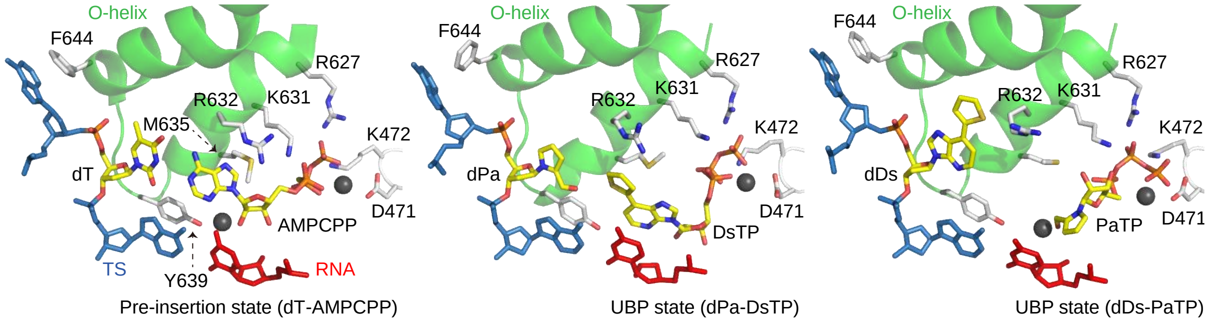

Supplementary Figure 3. Substrate recognition of UBPs and natural ATP at pre-insertion state. Different view from Figure 3, highlighting key interactions for substrate selection.

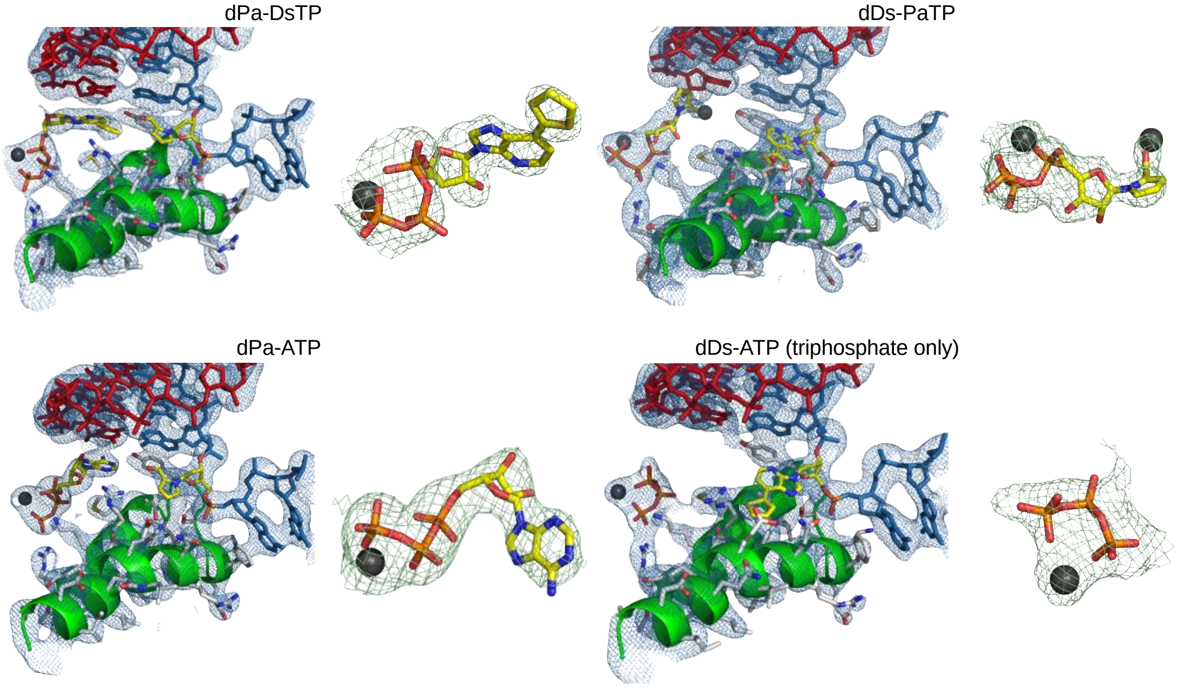

Supplementary Figure 4. Electron density maps of active site, RNA/DNA scaffold and bound substrate. Each electron density maps were generated using PHENIX composite omit map. All of maps were contoured at 1.2 σ. 2Fo-Fc and Fo-Fc maps were colored in blue and green, respectively.

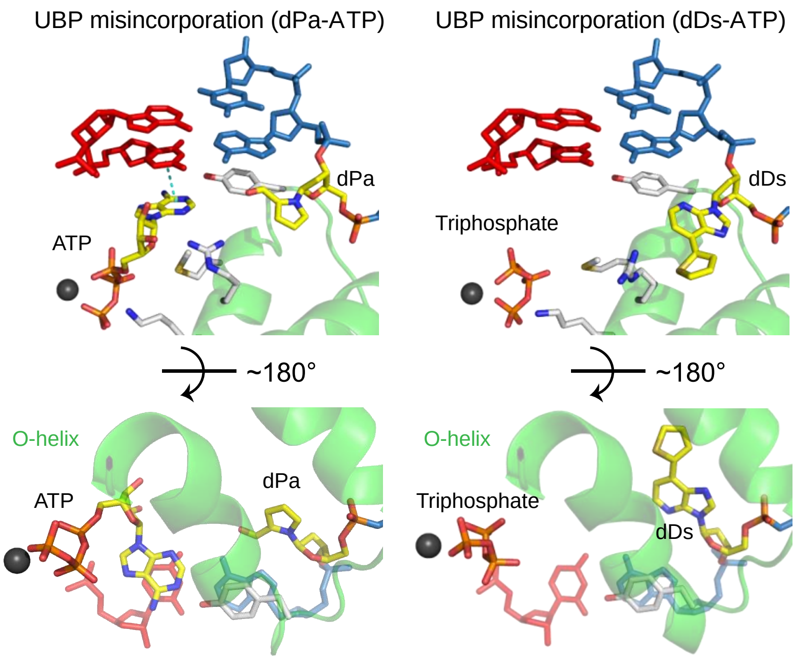

Supplementary Figure 5. Structures of distinct ATP binding against UBP templates at pre-insertion state. dPa–ATP and dDs–ATP structures (only density of triphosphate moiety was visualized and modeled). Coloring scheme is the same as Figure 3.

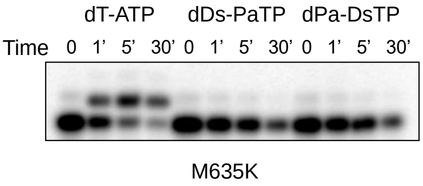

Supplementary Figure 6. Single incorporation transcription assay of T7 RNAP M635K mutant. Same experiment method was used in Fig. 4. Raw gel images for Fig. 4 and Supplementary Fig. 6 are shown in source data fig. 4.

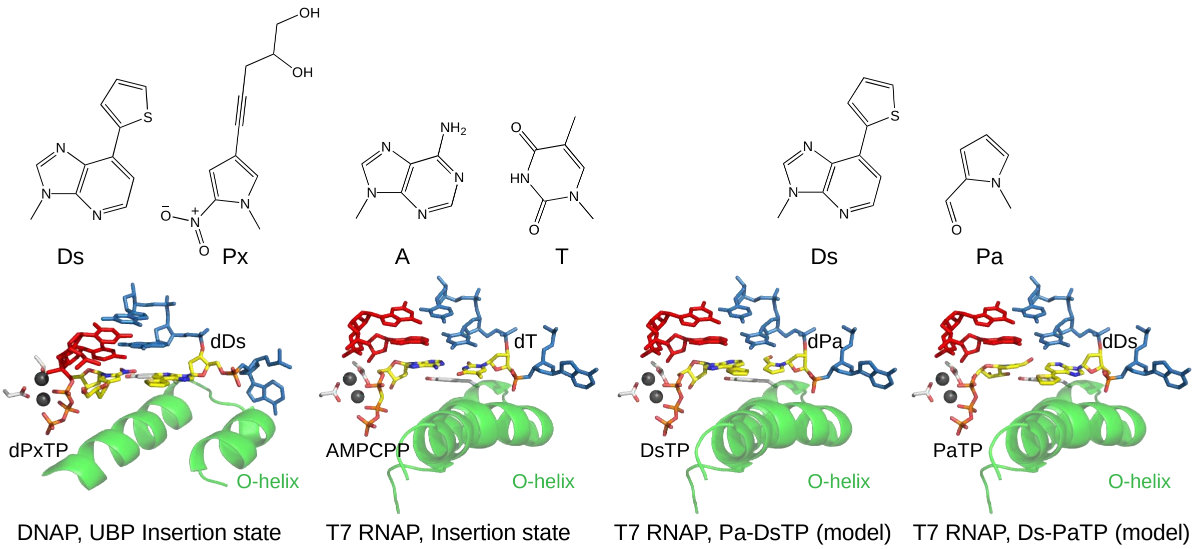

Supplementary Figure 7. Structural model of Ds–Pa T7 RNA polymerase at insertion state. Top panel: schematic representation of base pairs in structures. Bottom panel (from left to right): Insertion state with dDs–dPxTP in DNA polymerase (PDB code: 5NKL); insertion state with dT–AMPCPP in T7 RNA polymerase (PDB code :1S76); insertion state models of dPa–DsTP and dDs–PaTP in T7 RNA polymerase.
