# Supplementary material for: Structural basis of transcription recognition of a hydrophobic unnatural base pair by T7 RNA polymerase
Source: Nat Commun. 2023 Jan 13;14:195. doi: 10.1038/s41467-022-35755-8 (PMC9836923; doi:10.1038/s41467-022-35755-8)
Supplement: Supplementary file 2 — Description of Additional Supplementary Files [file 41467_2022_35755_MOESM2_ESM.pdf]

Supplementary Movie 1.

Structural modeling of UBP incorporation. Models of dT-ATP incorporation (left panel), dPa-DsTP incorporation (middle panel) and dDs-PaTP incorporation (right panel) were shown.
